# Development and validation of a social impact questionnaire for household food waste

**DOI:** 10.1016/j.mex.2023.102499

**Published:** 2023-11-25

**Authors:** Yayuk Farida Baliwati, Rian Diana, Drajat Martianto, Dadang Sukandar, Agung Hendriadi

**Affiliations:** a Department of Community Nutrition, Faculty of Human Ecology, IPB University, Bogor 16680, Indonesia; b Program of Nutrition Science, Graduate School, IPB University, Bogor 16680, Indonesia; c Department of Nutrition, Faculty of Public Health, Universitas Airlangga, Surabaya 60115, Indonesia; d Agroindustry Research Center, National Research and Innovation Agency, Jakarta 10340, Indonesia

**Keywords:** Explorative study, Consumer behavior, Food waste, Perceive, Reliability, Sustainable consumption, Social Impact Questionnaire for Household Food Waste

## Abstract

This exploratory study was conducted to develop and validate an instrument for measuring the social impact of food waste at the household level. A mixed methodology was employed, commencing with a qualitative approach through in-depth interviews and focus group discussions, to identify the social impact of food waste at the household level. This was followed by a quantitative assessment of the questionnaire's validity and reliability. The process involved seven experts for content validity testing, 150 households for face validity testing, and 202 households for reliability testing. The content validity revealed an I-CVI 0.86-1 and a kappa statistic 0.85-1. The face validity demonstrated I-FVI ≥ 0.90 and S-FVI ≥ 0.95. Additionally, the Cronbach's alpha for the reliability test was 0.743. The questionnaire proved to be a simple, valid, and reliable instrument for measuring the social impact of household food waste.

• Mixed method used in developing and validating the questionnaire.
• Content validity, face validity, and reliability testing to evaluate a newly developed instrument.

## Highlights

• Mixed method used in developing, validating, testing the reliability of the new social impacts of food waste questionnaire.
• Content validity, face validity, reliability testing to evaluate a newly developed instrument for food waste social impacts.
• A simple, valid, reliable questionnaire for measuring the social impact of household food waste for sustainable consumption.

## Graphical abstract

**Figure:**
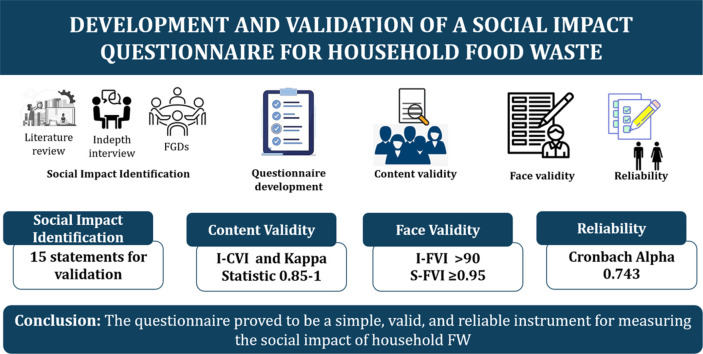

Specifications table

**Table:** 

Subject area:	Environmental Science
More specific subject area:	*Household Food Waste*
Name of your method:	*Social Impact Questionnaire for Household Food Waste*
Name and reference of original method:	*NA*
Resource availability:	*NA*

## Method details

Food waste (FW) occurs at the final stages of the food supply chain, including the retail, food service, household, and individual levels. Among these stages, household food waste is the main contributor to overall food waste, both globally and in Indonesia [1,2], and has significant environmental, economic, and social consequences [3].

Social impacts have a wide-ranging scope, covering environmental, economic, social, cultural, political, health, educational, psychological, and spiritual domains [4]. On the other hand, the human welfare ecology approach emphasizes that sustainable development must address both physical and social factors to improve the quality of life. Therefore, the social impact of food waste in this study encompasses all physical and social factors that society experiences [5]. Several studies have investigated the social impact of food waste reduction and redistribution programs, focusing on retail, food services, and corporate levels [6], [7], [8], [9]. Several studies have explored the social impact of food waste at the household level [10], [11], [12]. However, studies that quantitatively measure and validate the social impact of food waste at the household level are scarce. Thus, this study aimed to develop and validate an instrument to measure the social impact of FW at the household level.

**Fig. 1:**
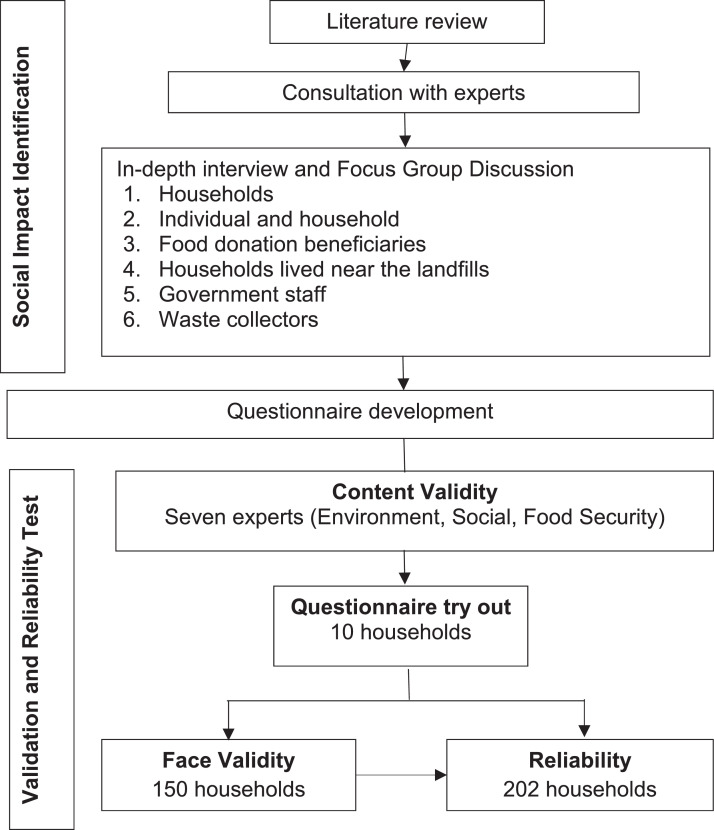
Steps in development and validation of household food waste social impact questionnaire.

The social impacts of household FW were measured in three stages. The first stage involved the identification of social impact; the second, the development of a questionnaire; and the third, the test for validation and reliability (Fig. 1). These stages were adapted from Fredline et al, Falcone et al, and Azimi et al [13], [14], [15].

1. Identification of Household FW Social Impact
  a. Literature review
    A literature review was conducted to establish a definition and scope of the social impact of FW. This review involved searching various sources, including peer-reviewed articles and gray literature (reports and books). Four experts were consulted to validate the results: two FW experts in environmental studies and two FW experts in social studies. The consultation findings served as the basis for the collection of primary data.
  b. Qualitative data were collected through in-depth interviews and focus-group discussions (FGD).
    The second phase, identifying social impacts, required the collection of primary data, including in-depth interviews and focus group discussions (FGD). The participation of the community or its stakeholders in the development of indicators that will be employed is crucial for identifying local issues considered significant by the community [13]. The following are the guidelines for conducting social impact identification FGDs: [1] participants were provided with a concise overview of the topic to be discussed prior to conducting the FGD; [2] participants were asked to discuss what impacts they felt related to FW, whether positive, negative, direct, indirect, short-term, or long-term; [3] participants were also encouraged to share their thoughts on the societal impacts relevant to household FW; and [4] the session concluded with closing remarks and conclusions.
    Qualitative data were gathered from various stakeholders, including communities and government officials. Data were collected through in-depth interviews with individuals or households that received food donations and waste collectors at the landfill site. FGDs were conducted with households living in urban and rural areas, those living near landfills, and government staff. Four FGDs were conducted, three with households and one with government staff. Qualitative data collection was conducted in Bogor Regency, West Java Province, Indonesia.
2. Questionnaire development
  The questionnaire was developed based on the identification of social impact. Fifteen social impacts of household FW were identified through a literature review and primary data collection. These effects were organized into questions and validated through content validation, face validation, and reliability testing.
  The results of the social impact identification in the initial stage indicated that FW has an impact on global warming. A review by Vanclay [16] on conceptualizing social impacts states that the environment or biophysical environment becomes a social impact category. Social impacts are related to the quality of the living environment or, in other words, the liveability of the neighborhood and workplace. This category contains many variables that have traditionally been considered in social and environmental impact assessment studies. Some of these variables are directly related to the physical environment or biophysical impacts. Therefore, food waste can cause global warming and become a part of the social impact.
  Based on the findings of social impact assessment in the early stages, food waste could have adverse effects on obesity, particularly among mothers. This is because mothers are more likely to consume leftover food at night (In-depth interviews). The unintended consequences of FW reduction interventions may negatively influence the risk of obesity. Social pressure to reduce FW may prompt an individual to ignore satiety cues and eat past the point of fullness, resulting in weight gain [17].
3. Validity and Reliability Testing
  a. Content Validity
    Content validity is determined by assessing how well each question item captures the entire domain or construct being measured (accurately reflecting the content being assessed), with expert judgment serving as the basis for the evaluation [18,19]. The process involves (1) preparation of content validity forms, (2) selection of experts with relevant knowledge, (3) implementation of content validity, (4) review of statements or questions by experts, (5) scoring each statement/question, and (6) calculation of the Content Validity Index (CVI) and kappa statistics.
    To evaluate content validity, seven experts, including three in the environment, two in social sciences, and two in food security, conducted qualitative and quantitative assessments. The panel comprised three academic staff members (one specializing in food security, another in food waste and social science, and a third in social science), an expert staff member from the Ministry of Environment and Forestry, a food security expert from the National Food Agency, and two solid waste experts from a non-governmental organization. The expert panel is selected based on their expertise in the fields of environment, social science, and food security related to food waste, with each member having more than 5 years of work experience. All experts provided quantitative assessments of relevance and qualitative assessments of clarity for all 15 statements.
    A comprehensive evaluation was performed by providing feedback on the relevance and clarity of all the statements or questions. In addition, experts provided input and suggestions for improving the content. A quantitative assessment was conducted using a 4-point Likert scale, rating the relevance of each question, with a 4-point Likert scale, with the following options: 1 = irrelevant, 2 = somewhat relevant, 3 = quite relevant, and 4 = very relevant.
    Content validity was determined based on the per-item Content Validity Index (I-CVI) values and multi-rater kappa statistics. The Content Validity Index was used to measure the content validity of the multi-item scales. The cut-off value for CVI is considered valid if CVI = 1 for 3–5 experts, CVI ≥ 0.83 for 6–8 experts, or CVI ≥ 0.78 for ≥ 9 experts [18]. The CVI was calculated by summing the number of experts who rated a question item as 3 (quite relevant) or 4 (very relevant) and dividing it by the total number of experts [19].
    Discrepancies in the assessments by multiple experts are expected. In the event of a discrepancy between the two experts, a third expert sought consultation. The final statements from the revised questionnaire were provided to the experts. To minimize the impact of disagreement among experts, a modified kappa statistic was calculated. The kappa statistic indicates the level of consistency in assessments between experts and the strength of agreement between observers. The strength of agreement is considered poor if the kappa statistic (K) < 0.00, slight if *K* = 0.00–0.20, fair if *K* = 0.21–0.40, moderate if *K* = 0.41–0.60, substantial if *K* = 0.61–0.80, and almost perfect if *K* = 0.81–1.00 [20]. According to Fleiss, a kappa value of ≤ 0.4 is considered poor agreement, while a value of 0.4–0.75 is considered good. If the kappa value is greater than 0.75, it is considered excellent [21]. To determine the kappa statistic, the probability of agreement (Pc) was calculated. All calculations were performed using Microsoft Excel 2019, and the following equations were used to calculate Pc and K [19].
    $$\begin{array}{ccc} \text{Pc} & = & [N!/A!\left(N-A\right)!*0,5^{N}\\ K & = & \left(I-\text{CVI}-\text{Pc}\right)/\left(1-\text{Pc}\right)\\ \text{Where:} & & \\ \text{Pc} & = & \text{Probability}\;\text{of}\;\text{change}\;\text{agreement}\\ \text{N} & = & \text{Total}\;\text{of}\;\text{experts}\\ \text{A} & = & \text{Total}\;\text{of}\;\text{experts}\;\text{who}\;\text{agree}\;\text{(relevant}\;\text{or}\;\text{clear)}\\ \text{K} & = & \text{Kappa}\;\text{statistic}\\ \text{I-CVI} & = & \text{Content}\;\text{Validity}\;\text{Index}\;\text{per}\;\text{item} \end{array}$$
  b. Face Validity
    Face validity was assessed after content validity to evaluate the level of importance, clarity, understandability, and simplicity of the language used. A try-out questionnaire was administered to 10 households prior to face validity. Face-to-face interviews were conducted with 150 households in Bogor district (105 urban and 45 rural), selected randomly from a sample of households that participated in waste collection during the initial phase of the study.
    The sample size for the face validity test was determined using a ratio of 10:1 with 10 participants for each item. This study uses 15 questions, resulting in a sample of 150 households. Aithal and Aithal stated that there is no fixed rule for calculating the sample size in questionnaire validation; however, it is recommended to use a large sample size to ensure a high response rate [22]. The ideal ratio for the sample size of the questionnaire validation study was 10:1 with 10 participants per item [23].
    Face validation was evaluated based on the level of importance, comprehensibility, and complexity of the language using a 5-point Likert scale. Each participant rated 15 statements, with the level of importance as 1 = not important, 2= slightly important, 3= undecided, 4= important, and 5= very important; the level of comprehension was 1= not easy to understand, 2= less easy to understand, 3= undecided, 4= easy to understand, and 5= very easy to understand; and the language complexity level was 1= complex, 2= slightly complex, 3= undecided, 4= simple, and 5= very simple. The results were calculated quantitatively to determine the Face Validity Index values for each statement item (I-FVI) and Scale-FVI (S-FVI). The S-FVI was the average of the overall I-FVI scores. For both online and face-to-face interviews, the recommended cutoff FVI value was above 0.8 [24].
    The participants involved in face validation were requested to provide their thoughts, opinions, and recommendations regarding the statements in the questionnaire. The interviewer inquired if there were any statements that were confusing, difficult to understand, used complex language, or were irrelevant or unimportant and needed to be removed.
  c. Reliability
    Reliability tests were conducted on the 202 households (137 urban and 65 rural) that participated in the household waste collection at the beginning of the study. Of the 215 households involved in FW data collection (initial phase), 202 completed the pilot questionnaire. Thirteen households dropped out because they had moved or were unavailable, despite three visits to their homes at different times.
    The reliability of the household FW social impact questionnaire was evaluated using Cronbach's alpha (α). The Cronbach's alpha value can be classified as unreliable (α ≤ 0), low internal consistency (0 < α ≤ 0.5), moderate internal consistency and reliable (0.5 < α < 0.7), sufficient internal consistency and reliable (α = 0.7), high internal consistency and reliable (0.7 < α < 0.9), some repeated/redundant items need to be eliminated (0.9 < α < 1), and perfect internal consistency (α = 1) [22]. Each participant was asked to express their opinion on the 15 statements in the questionnaire using a 5-point Likert scale: 1= strongly agree, 2= agree, 3= undecided, 4= disagree, and 5= strongly disagree.
    Pearson's product-moment correlation analysis was used to determine the validity of the items. Pearson's correlation analysis was performed by correlating the statement item scores with the total statement item scores. Statement items were considered valid if their correlation coefficient was greater than the r-table value based on the degree of freedom of the estimated variable, with a significance level of 5%. Pearson's correlation and Cronbach's alpha tests were conducted using the IBM SPSS 21.

## Validation methods

### Content validity

This study demonstrates that the questionnaire, which comprises 15 statements, is a valid instrument for measuring the social impact of FW at the household level. All statements were found to be relevant for measuring the social impact of household FW and had a high level of inter-rater agreement, with the I-CVI values ranging from to 0.86–1 and kappa statistic values of 0.85–1 for all items or statements (Table 1). The CVI value considered valid for to 6–8 expert sources was greater than 0.83 [18]. Additionally, the kappa statistic values of 0.81–1 indicated a very strong level of agreement between raters [20], and kappa values greater than 0.75 were considered to have an excellent level of agreement [21].

**Table 1:** I-CVI dan *kappa statistic* of each item for relevance.

Item	Number of experts who agree	I-CVI	Pc	Kappa statistic	Interpretation
1	7	1	0.008	1	Excellent
2	7	1	0.008	1	Excellent
3	7	1	0.008	1	Excellent
4	7	1	0.008	1	Excellent
5	6	0.86	0.055	0.85	Excellent
6	6	0.86	0.055	0.85	Excellent
7	6	0.86	0.055	0.85	Excellent
8	6	0.86	0.055	0.85	Excellent
9	7	1	0.008	1	Excellent
10	7	1	0.008	1	Excellent
11	7	1	0.008	1	Excellent
12	7	1	0.008	1	Excellent
13	7	1	0.008	1	Excellent
14	6	0.86	0.055	0.85	Excellent
15	6	0.86	0.055	0.85	Excellent

Content validation was carried out to evaluate the new instruments, ensure that all items in the instrument were important or relevant, and to eliminate items that were not related to the construct. Therefore, content validation should be conducted when developing new instruments [25]. An instrument is considered valid if it includes relevant components that accurately measure the construct. Instruments should include only a representative number of items to avoid redundancy and ensure accuracy [26].

Quantitatively, a questionnaire consisting of 15 statements was found to be a valid instrument for measuring the social impact of FW at the household level. However, 12 statements required revision to enhance comprehension and clarity (Table 2). Improvements were made to each statement after receiving feedback from an expert panel on the comprehension, clarity, and complexity of the language. Three statements did not need to be revised: (1) throwing edible food is a wasteful behavior; (2) sharing surplus food can increase social engagement (sharing meals with neighbors, friends, and others); and (3) throwing away edible food means throwing away money.

**Tabel 2:** Identification and revision of each statement in terms of language complexity and comprehension.

Statement	Comments from Expert Panel	Statement Revision
1. Throwing away food makes you feel guilty.	The word “edible” needs to be added “throw away edible food.”	Throwing away edible food makes you feel guilty.
2. Throwing away food makes you feel sinful.	The word “edible” needs to be added “throw away edible food.”	Throwing away edible food makes you feel sinful.
3. Leaving food uneaten or throwing food can set a bad example for children/others.	The phrase “leaving food uneaten” does not mean to be thrown away but can also be used for other purposes such as giving it to others.	Throwing edible food can set a bad example for children/others.
4. Food waste creates odor.	The word “odor” can be added to unpleasant odor/pollution. The phrase “unattended food waste for an extended period of time” should be added.	Unattended food waste for an extended period of time can cause an unpleasant odor.
5. Food waste can cause global warming.	The phrase “global warming” is difficult for the general public.	Food waste is contributing to global warming by increasing the earth's temperature.
6. Food waste is beneficial for plants (compost).	Not all food waste. The phrase “compost or compost raw materials” should be added.	Vegetable and fruit scraps can be processed into compost or compost raw materials.
	“”	
7. Food waste can be used as animal feed (chicken, duck, catfish, etc.).	The phrase “feed or feed ingredients” should be added.	Food waste can be used as animal feed of feed ingredients (chicken, duck, catfish, etc.).
8. Food waste can attract animals/pests.	The statement should include examples of animals/pests and “food waste that is left open” should be added.	Food waste that is left open can invite animals/pests (rats, flies, maggots, etc.).
9. Food waste can reduce food availability at home.	The phrase “throwing away edible food" should be added.	Throwing away edible food can reduce food availability at home.
10. Leftovers can increase people's consumption (leftovers are given to others).	The phrase “throwing away edible food” should be added.	Giving away surplus food or edible leftovers to others can increase their consumption.
	Surplus food should be able to meet the need of others food consumption	
11. Food waste can reduce food consumption at home (discarding edible food).	The statement requires further clarification as it is somewhat unclear and difficult to understand.	Throwing away edible food can reduce family consumption at home.
12. Leftover food can cause mother's obesity	The statement is unclear and difficult to understand.	Eating leftovers often at night can lead to obesity
	Not only mothers.	

Revisions to the statement were made after seven expert panels conducted content validation. The questionnaire was pilot-tested in 10 households. The respondents consisted of five men and five women aged 29–63 years (40% 40–59 years old, 30% < 40 years and > 60 years). Most participants were high school graduates (80%) employed as entrepreneurs, teachers, or housewives. A trial was conducted to assess the difficulty level of the questionnaire, focusing on its clarity, comprehension, and language complexity. No revisions were made to the questionnaire after the trial because all respondents had already understood all the statements in the questionnaire.

## Face validity

Table 3 shows that all statement items had excellent face validity. This can be seen from the values of I-FVI ≥ 0.90 and S-FVI ≥ 0.95, for all aspects. All statements were considered important, and the language used was simple and easy to understand. The recommended FVI cut-off value is above 0.8 [24].

**Table 3:** I-FVI of each item for importance, comprehension, and language complexity.

Item	Importance		Comprehension		Language Complexity		Interpretation
	Number of experts who agree	I-FVI	Number of experts who agree	I-FVI	Number of experts who agree	I-FVI	
1	150	1	149	0.99	149	0,99	Excellent
2	146	0.97	149	0.99	146	0,97	Excellent
3	146	0.97	148	0.99	148	0,99	Excellent
4	148	0.99	148	0.99	147	0,98	Excellent
5	147	0.98	148	0.99	149	0,99	Excellent
6	134	0.89	117	0.78	132	0,88	Excellent
7	146	0.97	146	0.97	146	0,97	Excellent
8	147	0.98	148	0.99	148	0,99	Excellent
9	148	0.99	146	0.97	148	0,99	Excellent
10	144	0.96	145	0.97	142	0,95	Excellent
11	143	0.95	139	0.93	142	0,95	Excellent
12	145	0.97	146	0.97	146	0,97	Excellent
13	143	0.95	139	0.93	142	0,95	Excellent
14	136	0.91	136	0.91	137	0,91	Excellent
15	138	0.92	138	0.92	135	0,90	Excellent
S-FVI	0.96		0.95		0.96	Excellent	

It is crucial that participants fill out the face validity of an instrument because experts cannot replace them. The participants' understanding and interpretation can affect the accuracy of the instrument used to evaluate the construct being studied. The clarity of the instructions and language ensures the absence of ambiguous or multiple interpretations. Comprehension of instructions and language indicates whether the statements or questions are easily understood by participants [24].

Face validity was conducted on 150 households, with 44.7% of respondents aged < 40 years and 52% aged between 40–60 years old. Additionally, 61.3% of the respondents had an education duration of < 9 years, whereas 29.3% had an education duration of 9–12 years. The face validity test showed that all statements were important, clear, and understandable. Statement 6 had the lowest I-FVI value compared to the other statements. Seventeen participants were unsure of the relationship between food waste and global warming (Item 6). Instead, they believe that plastic contributes to global warming. Meanwhile, three participants suggested that Item 15, which states that eating leftovers often at night can lead to obesity, requires clarification. Specifically, they noted that the effect of leftovers on obesity depends on what is consumed, with leftover fruits and vegetables not contributing to obesity.

### Reliability

Reliability testing was conducted on 202 households, with 42.6% of the respondents aged under 40 years old and 52.5% aged between 40–60 years old. Most respondents (91.5%) had an education level of ≤ 12 years.

Table 4 shows that all statements regarding the perceived social impact of household FW were valid (*p* < 0.001) and reliable (Cronbach's alpha > 0.7). Cronbach's alpha for this questionnaire was 0.743. Cronbach's alpha values between 0.7–0.9 indicate high internal consistency [22]. Additionally, Table 4 indicates that the Cronbach's alpha value of two statements (Items 2 and 14) would increase if they were removed, but the change would not significantly affect the Cronbach's alpha value.

**Tabel 4:** Item analysis and reliability of household FW social impact questionnaire.

Item	r-item	p	*Cronbach's Alpha*	*Cronbach's Alpha if item deleted*	Interpretation
1	0.437	0.000	0.757	0.746	Reliable
2	0.322	0.000	0.757	0.765	Reliability increased if item deleted
3	0.599	0.000	0.757	0.734	Reliable
4	0.510	0.000	0.757	0.742	Reliable
5	0.511	0.000	0.757	0.740	Reliable
6	0.502	0.000	0.757	0.747	Reliable
7	0.460	0.000	0.757	0.745	Reliable
8	0.549	0.000	0.757	0.740	Reliable
9	0.592	0.000	0.757	0.737	Reliable
10	0.476	0.000	0.757	0.744	Reliable
11	0.491	0.000	0.757	0.748	Reliable
12	0.530	0.000	0.757	0.739	Reliable
13	0.537	0.000	0.757	0.738	Reliable
14	0.496	0.000	0.757	0.759	Reliability increased if item deleted
15	0.541	0.000	0.757	0.743	Reliable

This study found that the questionnaire was a simple, valid, and reliable instrument for measuring the social impact of household FW. Validation indicates the relevance between the instrument and the construct to be assessed, whereas reliability indicates the consistency of results from repeated use of the instrument [26]. This questionnaire is the first instrument used to measure the social impact of FW at the household level. It was developed and validated in Bogor Regency and covers both urban and rural areas with a predominantly Sundanese population. Additionally, a moderate sample size was used for reliability testing. Further validation of the reliability of the social impact of household FW is needed in different regions with varying cultures and geographical conditions, and a larger sample size.

This study developed and evaluated the validity and reliability of the social impact of FW at the household level. Future studies could measure the overall social impact of FW on retail, food services, and households by employing the methodology used in this study.

## Ethics statements

This study was approved by the Human Research Ethics Committee of Bogor Agricultural University (Number: 747/IT3.KEPMSM-IPB/SK/2022). Participants were informed of the study's objectives and benefits and were provided with the right to withdraw at any time without consequences. Confidentiality was maintained throughout, and informed consent was obtained from participants before the interview. All data were treated as such and no sanctions were imposed on those who chose to withdraw.

## Acknowledgments

The authors would like to thank the Bogor Regency government and all participants involved in this study.

## Funding

This work was supported by the Neys-van Hoogstraten Foundation and IPB University [grant numbers: 01/NHF-IPB/2022].

*Please declare any financial interests/personal relationships which may be considered as potential competing interests here.*

## CRediT authorship contribution statement

**Yayuk Farida Baliwati:** Writing – original draft, Conceptualization. **Rian Diana:** Writing – original draft, Validation. **Drajat Martianto:** Writing – review & editing, Supervision. **Dadang Sukandar:** Methodology, Writing – review & editing. **Agung Hendriadi:** Writing – review & editing, Supervision.

## Declaration of Competing Interest

The authors declare that they have no known competing financial interests or personal relationships that could have appeared to influence the work reported in this paper.

## Data Availability

Data will be made available on request.

## Appendix Supplementary materials

Social Impact of HH Food Waste Questionnaire_Indonesia
