# Supplementary material for: Development and validation of a social impact questionnaire for household food waste
Source: MethodsX. 2023 Nov 25;11:102499. doi: 10.1016/j.mex.2023.102499 (PMC10697991; doi:10.1016/j.mex.2023.102499)
Supplement: Supplementary file 1 [file mmc1.zip › mmc1/Social Impact of HH Food Waste Questionnaire_English.pdf]

HOUSEHOLD FOOD WASTE SOCIAL IMPACT QUESTIONNAIRE

Below are a number of statements regarding the social impact of household food waste. Please read each one and indicate to what extent you agree or disagree with each statement by putting a check mark (√).

**STATEMENTS**		**OPINION**				
**No.**	**Statements**	**Strongly Agree**	**Agree**	**Undecided**	**Disagree**	**Strongly Disagree**
1.	Throwing edible food makes you feel guilty					
2.	Throwing edible food makes you feel sinful					
3.	Throwing edible food is a wasteful behavior					
4.	Throwing edible food can set a bad example for children/others					
5.	Unattended food waste for an extended period of time can cause an unpleasant odor					
6.	Food waste is contributing to global warming by increasing the earth's temperature					
7.	Vegetable and fruit scraps can be processed into compost or compost raw materials					
8.	Food waste can be used as animal feed of feed ingredients (chicken, duck, catfish, etc.)					
9.	Food waste that is left open can invite animals/pests (rats, flies, maggots, etc.)					
10	Sharing surplus food can increase social engagement (sharing meals with neighbors, friends, and others)					
11	Throwing away edible food can reduce food availability at home					
12	Throwing away edible food means throwing away money					
13	Giving away surplus food or edible leftovers to others can increase their consumption					
14	Throwing away edible food can reduce family consumption at home					
15	Eating leftovers often at night can lead to obesity					
