# Supplementary material for: Development and validation of a social impact questionnaire for household food waste
Source: MethodsX. 2023 Nov 25;11:102499. doi: 10.1016/j.mex.2023.102499 (PMC10697991; doi:10.1016/j.mex.2023.102499)
Supplement: Supplementary file 1 [file mmc1.zip › mmc1/Social Impact of HH Food Waste Questionnaire_Indonesia.pdf]

KUESIONER DAMPAK SOSIAL FOOD WASTE RUMAH TANGGA

Berikut sejumlah pernyataan terkait dampak sosial *food waste* rumah tangga. Silahkan baca setiap pernyataan dan isikan pendapat anda setuju atau tidak setuju dengan pernytaan tersebut dengan memberikan tanda ceklist (√)

**PERNYATAAN**		**PENDAPAT**				
**No.**	**Pernyataan**	**Sangat Setuju**	**Setuju**	**Ragu-ragu**	**Kurang setuju**	**Tidak setuju**
1.	Menyisakan atau membuang makanan yang masih layak dimakan membuat anda **merasa bersalah**					
2.	Menyisakan atau membuang makanan yang masih layak dimakan membuat anda **merasa berdosa**					
3.	Menyisakan dan membuang makanan layak konsumsi merupakan hal yang **mubazir**					
4.	Membuang makanan masih layak dimakan dapat menjadi contoh yang tidak baik untuk anak/ orang lain					
5.	Sisa makanan/ sampah makanan yang dibiarkan beberapa lama dapat menimbulkan **bau kurang sedap**					
6.	Sisa makanan/ sampah makanan dapat menyebabkan **suhu bumi lebih panas** sehingga meningkatkan **pemanasan global**					
7.	Sisa sayuran dan buah dapat diolah menjadi **kompos atau bahan baku kompos**					
8.	Sisa makanan/ sampah makanan dapat diolah menjadi **pakan atau bahan baku pakan ternak** (ayam, bebek, lele, dll)					
9.	Sisa/sampah makanan yang dibiarkan terbuka dapat **mengundang binatang/hama** (tikus, lalat, belatung, dll)					
10	Membagikan kelebihan makanan dapat **meningkatkan kebersamaan/keakraban** (makan bersama tetangga/teman, dll)					
11	Membuang makanan yang layak dimakan dapat **menurunkan ketersediaan pangan di rumah**					
12	Menyisakan atau membuang makanan sama dengan **membuang uang**					
13	Memberikan kelebihan makanan atau sisa makanan yang masih layak dimakan dapat **meningkatkan konsumsi orang lain**					
14	Membuang makanan yang layak dimakan dapat **menurunkan konsumsi keluarga di rumah**					
15	Sering mengonsumsi makanan sisa di malam hari dapat **menyebabkan kegemukan**					
